# Association of *CYP19A1* rs28757157 polymorphism with lung cancer risk in the Chinese Han population

**DOI:** 10.1186/s12957-022-02868-9

**Published:** 2022-12-16

**Authors:** Chan Zhang, Yujing Cheng, Wanlu Chen, Qi Li, Run Dai, Yajie Wang, Tonghua Yang

**Affiliations:** 1 Department of Blood Transfusion, The First People’s Hospital of Yunnan Province, Kunming, 650032 Yunnan China; 2 Department of Hematology, The First People’s Hospital of Yunnan Province, Xishan District, #157 Jinbi Road, Kunming, 650032 Yunnan China

**Keywords:** *CYP19A1*, Polymorphisms, Lung cancer, Case–control study, Chinese Han population

## Abstract

**Background:**

Lung cancer is the leading cause of cancer death globally. Recent studies have revealed that *CYP19A1* gene plays a crucial role in cancer initiation and development. The aim of this study was to assess the association of *CYP19A1* genetic polymorphisms with the risk of lung cancer in the Chinese Han population.

**Methods:**

This study randomly recruited 489 lung cancer patients and 467 healthy controls. The genotypes of four single nucleotide polymorphisms (SNPs) of the *CYP19A1* gene were identified by the Agena MassARRY technique. Genetic model analysis was used to assess the association between genetic variations and lung cancer risk. Odds ratios (ORs) and 95% confidence intervals (CIs) were calculated to evaluate the effect of four selected SNPs on lung cancer risk.

**Results:**

*CYP19A1* rs28757157 might contribute to an increased risk of lung cancer (*p* = 0.025, OR = 1.30, 95% CI 1.03–1.64). In stratified analysis, rs28757157 was associated with an increased cancer risk in the population aged under 60 years, females, smokers, and drinkers. Besides, rs3751592 and rs59429575 were also identified as risk biomarkers in the population under 60 years and drinkers. Meanwhile, a relationship between an enhanced risk of squamous cell carcinoma and rs28757157 was found, while the rs3751592 CC genotype was identified as a risk factor for lung adenocarcinoma development.

**Conclusions:**

This study has identified revealed that the three SNPs (rs28757157, rs3751592, and rs59429575) of *CYP19A1* are associated with lung cancer in the Chinese Han population. These findings will provide theoretical support for further functional studies of *CYP19A1* in lung cancer.

**Supplementary Information:**

The online version contains supplementary material available at 10.1186/s12957-022-02868-9.

## Background

Lung cancer is a kind of malignant tumor with high morbidity and mortality [1]. In China, this malignant tumor has the highest mortality rate, accounting for about 25% of cancer-related deaths in the world [2]. At present, many risk factors are found to increase the risk of lung cancer. Among them, smoking seems to be strongly associated with lung cancer risk [3]. However, a new research has shown that worldwide, 15–20% of men with lung cancer are non-smokers while over 50% of women with lung cancer are non-smokers [4], indicating the importance of other risk factors such as exogenous air pollution, environmental, and genetic factors. According to the latest statistics, genetic factors have been identified to be robustly associated with lung cancer [5]. If the family history of lung cancer is from a first-degree relative, the risk increases by 2–4 times even after controlling for smoking history [6].

Lung cancer is the leading cause of cancer mortality worldwide, in which women are less than half as likely to die of lung cancer as men [1]. Lung cancer in non-smokers tends to be more common in females [4]. These findings have drawn attention to investigate the effects of estrogen on lung cancer risk. It has been reported that both estrogen receptor and aromatase are present in human lung tumors [7, 8]. These results suggest that estrogen may play a role in the biological behavior of human lung cancer.

Cytochrome p450 (CYP450) enzymes are pivotal for biological homeostasis. CYP450 enzymes also play a key role in the metabolism of many endogenous substrates and exogenous carcinogens as well as aromatic and heterocyclic amines. They then covalently combine with DNA to form DNA adducts, which in turn cause cancer [9, 10]. The CYP450 family 19, subfamily A, and polypeptide 1 (*CYP19A1*) gene encodes aromatase, which is a member of the CYP450 superfamily and a key enzyme in oestradiol biosynthesis. Mutations in the *CYP19A1* gene can result in either increased or decreased aromatase activity [11], and aromatase plays an important role in lung cancer [12]. This suggests that *CYP19A1* genetic variations may indirectly affect the occurrence of lung cancer, but the exact mechanism is unclear. At the same time, many works of literature have reported an inseparable relationship between the genetic variant of *CYP19A1* and lung-related diseases, including lung cancer [13]. Previously, *CYP19A1* rs3764221 has been studied to be significantly associated with the multicentric development of lung adenocarcinomas [13]. Moreover, *CYP19A1* rs727479 is also significantly associated with the incidence of lung cancer [14]. However, there are still a large number of single-nucleotide polymorphisms (SNPs) in *CYP19A1* whose association with lung cancer risk has not been reported.

Based on Han Chinese in Beijing (CHB) population in 1000 genome database (http://www.internationalgenome.org/) and the dbSNP database (http://www.bioinfo.org.cn), four SNPs (rs28757157 (NG_007982.1:g.90395G > C), rs3751592 (NG_007982.1:g.29218A > G), rs3751591 (NG_007982.1:g.29086 T > C), and rs59429575 (NG_007982.1:g.28719G > A)) in *CYP19A1* with the minor allele frequency more than 5% were randomly selected. These SNPs in this study have been reported in the genome-wide association studies (GWAS) chips of published GWAS studies about testicular germ cell tumor and breast cancer [15, 16], but not lung cancer. Here, this study aimed to investigate the association between these four SNPs in the *CYP19A1* gene and lung cancer susceptibility through a case–control study.

## Methods

### Participants

In order to ensure the accuracy and credibility of the research results, we used G * Power 3.1.9.7 software (https://stats.idre.ucla.edu/other/gpower/) to estimate the sample size before we planned to conduct this study. The specific parameters we set were as follows: effect size *d* = 0.2; *α* error probability = 0.05; and power (1-β error probability) = 80%. This calculation produced a sample of at least 412 cases and 412 controls. Here, we recruited 489 cases and 467 controls in this study, larger than the total sample size recommended by G * Power. In the study, we recruited 489 pathologically confirmed lung cancer patients from Xuanwei City, Yunnan. All cases were diagnosed as lung cancer by histological examination according to the World Health Organization tumor classification system and confirmed by two independent pathologists. The exclusion criteria for patients were as follows: (1) history of other tumors; (2) family history of lung cancer; (3) chemotherapy or radiotherapy treatment; (4) hypertension, diabetes mellitus, or any endocrine metabolic diseases; and (5) other lung diseases. The control group was composed of 467 healthy subjects who were volunteer blood donors from the same city as the cases. Controls with a history of any cancers, other endocrine metabolic diseases, or other lung diseases should be excluded. Eligible study participants were screened by completing a specialized questionnaire, which included demographic characteristics, disease history, lung status, and family history of other types of tumors. All participants were of Chinese Han ancestry from northwest China. The research protocol according to the Helsinki Declaration was conducted with the approval of the First People’s Hospital of Yunnan Province Ethics Committee, and written informed consent from all subjects was attained.

### SNP selection

Four SNPs (rs28757157 (NG_007982.1:g.90395G > C), rs3751592 (NG_007982.1:g.29218A > G), rs3751591 (NG_007982.1:g.29086 T > C), and rs59429575 (NG_007982.1:g.28719G > A)) in *CYP19A1* were randomly selected based on the following: (1) the variations of *CYP19A1* through the e!GRCh37 (http://asia.ensembl.org/Homo_sapiens/Info/Index) database in the CHB and CHS population; (2) Hardy–Weinberg Equilibrium (HWE) > 0.01, minor allele frequency (MAF) > 0.05, and min genotype > 75% using Haploview software; (3) combined MassARRAY primer design software, HWE > 0.05, MAF > 0.05, and the call rate > 95% in our study population; and (4) a MAF > 0.05 based on the database of 1000 genome (http://www.internationalgenome.org/) and dbSNP (http://www.bioinfo.org.cn) databases.

### SNP genotyping

Genomic DNA was extracted from collected peripheral blood samples using a DNA purification extraction kit (GoldMag Xi’an, China). The concentration and purity of DNA were determined quantitatively by an ultraviolet spectrophotometer (Nanodrop 2000, Thermo, USA). Multiplexed SNP MassEXTEND assay was designed with the Agena Bioscience Assay Design Suite software, version 3.0 (Agena Bioscience, USA). SNP genotyping was conducted utilizing the MassARRAY platform (Agena Bioscience, USA). The principle of MassARRAY is matrix-assisted laser desorption/ionization (MALDI) time-of-flight (TOF) mass spectrometry (MS). First, a locus-specific PCR reaction was performed, followed by a locus-specific primer extension reaction (iPLEX assay), in which oligonucleotide primers were annealed directly upstream of the polymorphism of genotyping. In the iPLEX assay, primers and amplified target DNA were incubated with a large number of modified dideoxynucleotide terminators. The primer extension is made according to the sequence of mutation sites and is a single complementary mass-modifying base. The quality of the extended primers was determined by MALDI-TOF mass spectrometry. The quality of the primers indicates the sequence, therefore, the allele present at the polymorphic locus of interest. Using MALDI-TOF mass spectrometry, SNP alleles could be identified with different qualities of extended primers [17, 18]. Data processing was carried out with Agena Bioscience TYPER software, version 4.0 (Agena Bioscience, San Diego, CA, USA) [19]. A 10% randomly selected samples were re-analyzed with 100% consistency for quality control.

### Statistical analysis and bioinformatics analysis

SPSS software (SPSS 22.0, USA) and Microsoft Excel were used for statistical analysis. Continuous variables were evaluated for normality using the Kolmogorov–Smirnov test. Continuous variables (age and body mass index (BMI)) with non-normal distribution as median with interquartile range (IQR) were compared using the Mann–Whitney *U* test. The differences in gender, smoking, and drinking distribution between the case and control groups were determined by the *χ*^2^ test. The *χ*^2^ test was used to determine whether individual polymorphisms were in HWE. In addition, *χ*^2^ test was used to detect the difference in allele and genotype frequencies between cases and controls. The SNPStats software (https://www.snpstats.net/start.htm?q=snpstats/start.htm) was adopted to define the relationship between polymorphisms and the risk of lung cancer in the Chinese Han population in different genetic model analyses (genotype, dominant, recessive, and additive models). Logistic regression analysis was used to calculate odds ratios (ORs) and 95% confidence intervals (CIs) to evaluate the relationship of four selected SNPs with lung cancer risk [20–22]. Binary logistic regression was used for the two SNP interactions associated with lung cancer susceptibility. The *p* < 0.05 was considered statistically significant in all tests. The functionality of candidate SNPs was annotated using the HaploReg v4.1 (https://pubs.broadinstitute.org/mammals/haploreg/haploreg.php), RegulomeDB (https://regulome.stanford.edu/regulome-search/), and QTLbase (http://www.mulinlab.org/qtlbase/index.html) databases.

In multifactor dimensionality reduction (MDR) analysis, multilocus genotypes were classified into high- and low-risk groups. With this method, multidimensional genotype variables were transformed into single-dimensional ones [23]. In order to explore the association of high-order SNP-SNP interactions with the susceptibility to lung cancer, we used the MDR method including cross-validation and permutation-test procedures. Cross-validation could minimize the possibility of false-positive results by dividing the data into a training set and a testing set and repeating each part of the data. Balanced accuracy was used to assess model quality. The overall best model with the greatest accuracy in the testing data was selected. The cross-validation consistency (CVC) provided a list of the number of cross-validation intervals in which a particular model was found. The permutation testing indicated the cross-validation consistency and the prediction error are statistically significant at the 0.001 level. This indicates that among 1000 permuted datasets, no best models had a cross-validation consistency or a prediction error of the same magnitude as was observed for the original dataset. Higher numbers indicated more robust results. A permutation test was used to assess the significance of the best model [24]. The optimal *CYP19A1* SNP-SNP interaction model for lung cancer susceptibility was performed through MDR 3.0.2 software.

## Results

### Study population

In this study, 489 lung cancer patients (337 males and 152 females) was involved as well as 467 healthy controls (326 males and 141 females). The median (IQR) ages of cases and controls were 61.00 (56.00–65.00) years old and 61.00 (55.00–65.00) years old, respectively (Table 1). In addition, the characteristics of the study population were collected for subsequent studies, including BMI, smoking, and drinking history, pathological type, pathological stage, and lymph node metastasis (LNM). There was no significant difference in age, gender, BMI, smoking, and drinking between the case group and the control group (*p* > 0.05).

**Table 1:** Characteristics of the study population

Characteristics	Cases	Controls	*p*
**Total**	489	467	
**Age, years**			0.379^**†**^
Median (IQR)	61.00 (56.00–65.00)	61.00 (55.00–65.00)	
≤ 60	218 (44.6%)	202 (43.3%)	
> 60	271 (55.4%)	265 (56.7%)	
**Gender**			0.765^‡^
Male	337 (68.9%)	326 (69.8%)	
Female	152 (31.1%)	141 (30.2%)	
**BMI, kg/m**^**2**^			0.592^**†**^
Median (IQR)	22.86 (20.42–24.94)	22.86 (19.84–25.26)	
< 24	307 (62.8%)	290 (62.1%)	
≥ 24	182 (37.2%)	177 (37.9%)	
**Smoking**			0.944^‡^
Yes	246 (50.3%)	236 (50.5%)	
No	243 (49.7%)	231 (49.5%)	
**Drinking**			0.371^‡^
Yes	144 (29.4%)	150 (32.1%)	
No	345 (70.6%)	317 (67.9%)	
**Pathological type**			
Squamous cell carcinoma	132 (27.0%)		
Adenocarcinoma	187 (38.2%)		
Absence	170 (34.8%)		
**Stage**			
I-II	82 (16.8%)		
III-IV	250 (51.1%)		
Absence	157 (32.1%)		
**LNM**			
Negative	84 (17.2%)		
Positive	213 (43.6%)		
Absence	192 (39.3%)		

*IQR* Interquartile range, *BMI* Body mass index, *LNM* Lymph node metastasis

^**†**^*p* values were calculated from Mann–Whitney *U* test

^‡^*p* values were calculated from two-sided *χ*^2^ test

### Genetic analyses of the selected SNPs with the risk of lung cancer

**Fig. 1:**
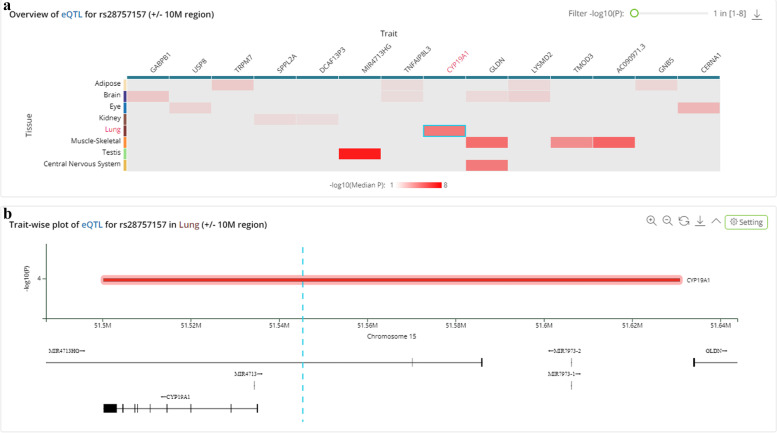
Overview of eQTL for rs28757157 (**a**) and trait-wise plot of eQTL for rs28757157 in the lung (**b**)

Four SNPs in *CYP19A1* were genotyped among subjects. The representative spectrum of each SNP is displayed in Supplemental Fig. 1. The basic information about all candidate SNPs is listed in Table 2. All SNPs are located on chromosome 15 and in the different positions of the *CYP19A1* gene. The deviation of Hardy–Weinberg equilibrium in the control group was evaluated, and the results showed that the candidate SNPs all met the expected *p* value (*p* > 0.05), and satisfied further study. In addition, under the allele model, there was a significant difference in the allele distribution of rs28757157 between the lung cancer cases (0.215) and healthy controls (0.174), and rs28757157 T allele might contribute to an increased risk of lung cancer (*p* = 0.025, OR = 1.30, 95% CI 1.03–1.64). Functional prediction of SNPs was conducted in HaploReg v4.1 and RegulomeDB databases to explore their regulatory effect. The results showed that four SNPs exhibited potential biological functions in gene regulation. Based on QTLbase database, the genotypes of *CYP19A1* rs28757157 (*p* = 6.610e − 5) were related to the mRNA expression of *CYP19A1* in the lungs (Fig. 1).

**Table 2:** Basic information of candidate SNPs *CYP19A1*

SNP ID	Chr	Position	Alleles A/B	MAF		*p*-HWE^**†**^	Heterozygosity	*p*^‡^	OR (95%CI)	HaploReg	RegulomeDB
				Case	Control						
rs28757157	Chr15	51,253,204	T/C	0.215	0.174	0.144	0.309	**0.025**	1.30 (1.03–1.64)	Enhancer histone marks, motifs changed, selected eQTL hits	TF binding + DNase peak
rs3751592	Chr15	51,314,381	C/T	0.133	0.112	0.999	0.199	0.172	1.21 (0.92–1.59)	Promoter histone marks, enhancer histone marks, DNAse, motifs changed, selected eQTL hits	TF binding + DNase peak
rs3751591	Chr15	51,314,513	G/A	0.137	0.133	0.840	0.235	0.798	1.04 (0.80–1.35)	Promoter histone marks, enhancer histone marks, DNAse, motifs changed	TF binding + DNase peak
rs59429575	Chr15	51,314,880	T/C	0.172	0.139	0.248	0.228	0.052	1.28 (1.00–1.64)	Promoter histone marks, enhancer histone marks, DNAse, proteins bound, motifs changed	TF binding or DNase peak

*SNP* single-nucleotide polymorphism, *MAF* minor allele frequency, *OR* odds ratio, *95%CI* 95% confidence interval

Bold values indicate statistical significance (*p* < 0.05)

^†^HWE *p* values were calculated from two-sided *χ*^2^ test

^‡^*p* values were calculated from two-sided *χ*^2^ test

Under four genetic models, the relationship between *CYP19A1* polymorphisms and the risk of lung cancer is listed in Table 3. Our results revealed an association between rs28757157 and increased risk of lung cancer in the genotype (*p* = 0.034, OR = 1.43, 95% CI 1.09–1.88), dominant (*p* = 0.011, OR = 1.41, 95% CI 1.08–1.85), and additive (*p* = 0.021, OR = 1.34, 95% CI 1.04–1.71) models.

**Table 3:** Analysis of the association between *CYP19A1* polymorphisms and risk of lung cancer

SNP ID	Model	Genotype	Control	Case	Crude		Adjusted	
					OR (95%CI)	*p*	OR (95%CI)	*p*
rs28757157	Genotype	C/C	313 (67.2%)	276 (59%)	1	**0.030**	1	**0.034**
		T/C	144 (30.9%)	183 (39.1%)	1.44 (1.10–1.89)		1.43 (1.09–1.88)	
		T/T	9 (1.9%)	9 (1.9%)	1.13 (0.44–2.90)		1.09 (0.42–2.79)	
	Dominant	C/C	313 (67.2%)	276 (59%)	1	**0.009**	1	**0.011**
		T/C-T/T	153 (32.8%)	192 (41%)	1.42 (1.09–1.86)		1.41 (1.08–1.85)	
	Recessive	C/C-T/C	457 (98.1%)	459 (98.1%)	1	0.990	1	0.910
		T/T	9 (1.9%)	9 (1.9%)	1.00 (0.39–2.53)		0.95 (0.37–2.43)	
	Additive	–-	–-	–-	1.35 (1.05–1.72)	**0.017**	1.34 (1.04–1.71)	**0.021**
rs3751592	Genotype	T/T	368 (78.8%)	374 (76.5%)	1	0.150	1	0.170
		C/T	93 (19.9%)	100 (20.4%)	1.06 (0.77–1.45)		1.06 (0.77–1.45)	
		C/C	6 (1.3%)	15 (3.1%)	2.46 (0.94–6.41)		2.40 (0.92–6.27)	
	Dominant	T/T	368 (78.8%)	374 (76.5%)	1	0.390	1	0.400
		C/T-C/C	99 (21.2%)	115 (23.5%)	1.14 (0.84–1.55)		1.14 (0.84–1.55)	
	Recessive	T/T-C/T	461 (98.7%)	474 (96.9%)	1	0.056	1	0.064
		C/C	6 (1.3%)	15 (3.1%)	2.43 (0.94–6.32)		2.37 (0.91–6.18)	
	Additive	–-	–-	–-	1.20 (0.92–1.56)	0.180	1.19 (0.91–1.56)	0.200
rs3751591	Genotype	A/A	348 (75%)	364 (74.7%)	1	0.820	1	0.800
		G/A	109 (23.5%)	113 (23.2%)	0.99 (0.73–1.34)		0.99 (0.73–1.34)	
		G/G	7 (1.5%)	10 (2%)	1.37 (0.51–3.63)		1.38 (0.52–3.69)	
	Dominant	A/A	348 (75%)	364 (74.7%)	1	0.930	1	0.920
		G/A-G/G	116 (25%)	123 (25.3%)	1.01 (0.76–1.36)		1.02 (0.76–1.36)	
	Recessive	A/A-G/A	457 (98.5%)	477 (98%)	1	0.520	1	0.510
		G/G	7 (1.5%)	10 (2%)	1.37 (0.52–3.63)		1.39 (0.52–3.69)	
	Additive	–-	–-	–-	1.04 (0.80–1.35)	0.800	1.04 (0.80–1.35)	0.790
rs59429575	Genotype	C/C	348 (74.7%)	345 (70.5%)	1	0.110	1	0.120
		T/C	106 (22.8%)	120 (24.5%)	1.14 (0.85–1.54)		1.14 (0.85–1.55)	
		T/T	12 (2.6%)	24 (4.9%)	2.02 (0.99–4.10)		1.99 (0.98–4.05)	
	Dominant	C/C	348 (74.7%)	345 (70.5%)	1	0.150	1	0.150
		T/C-T/T	118 (25.3%)	144 (29.4%)	1.23 (0.93–1.64)		1.23 (0.92–1.64)	
	Recessive	C/C-T/C	454 (97.4%)	465 (95.1%)	1	0.056	1	0.061
		T/T	12 (2.6%)	24 (4.9%)	1.95 (0.96–3.95)		1.93 (0.95–3.91)	
	Additive	–-	–-	–-	1.25 (0.99–1.59)	0.063	1.25 (0.98–1.59)	0.066

*p* values were calculated from logistic regression without and with adjustments for age, gender, BMI, smoking, and drinking

Bold values indicate statistical significance (*p* < 0.05)

### Stratification analyses by demographic characteristics

In addition, we conducted a stratified analysis by demographic characteristics (age, gender, BMI, smoking, and drinking) to explore the risk effects of these SNPs in specific groups, as shown in Table 4. The results of age stratification indicated that rs28757157 (genotype: *p* = 0.018, OR = 1.83; dominant: *p* = 0.005, OR = 1.82; and additive: *p* = 0.006, OR = 1.78), rs3751592 (genotype: *p* = 0.032, OR = 1.87; dominant: *p* = 0.010, OR = 1.93; and additive: *p* = 0.009, OR = 1.81), and rs59429575 (genotype: *p* = 0.047, OR = 1.71; dominant: *p* = 0.014, OR = 1.75; and additive: *p* = 0.016, OR = 1.57) were associated with an increased susceptibility to lung cancer in people aged under 60 years. Moreover, rs28757157 exerted a risk role in the development of lung cancer among females in the dominant (*p* = 0.033, OR = 1.76), and additive (*p* = 0.036, OR = 1.70) models. In smokers, rs28757157 (dominant: *p* = 0.031, OR = 1.55; and additive: *p* = 0.042, OR = 1.46) might confer to a higher risk for the occurrence of lung cancer. In addition, rs28757157 (genotype: *p* = 0.033, OR = 2.03; dominant: *p* = 0.009, OR = 2.04; and additive: *p* = 0.010, OR = 1.99) and rs59429575 (dominant: *p* = 0.044, OR = 1.75; and additive: *p* = 0.044, OR = 1.63) were related to an increased risk of lung cancer in drinkers, whereas rs3751592 (*p* = 0.023, OR = 3.31) was identified as a genetic risk factor for lung cancer susceptibility in non-drinkers. However, no significant correlation between *CYP19A1* polymorphisms and lung cancer risk after stratification by BMI was found.

**Table 4:** Stratification analyses by demographic characteristics for the association between *CYP19A1* polymorphisms and the risk of lung cancer

Group/SNPs	Model	Genotype	Control	Case	OR (95%CI)	*p*	Control	Case	OR (95%CI)	*p*
**Age, years**	> 60						≤ 60			
rs28757157	Genotype	C/C	172 (65.2%)	156 (60.5%)	1	0.490	141 (69.8%)	120 (57.1%)	1	**0.018**
		T/C	84 (31.8%)	94 (36.4%)	1.25 (0.86–1.82)		60 (29.7%)	89 (42.4%)	1.83 (1.20–2.79)	
		T/T	8 (3%)	8 (3.1%)	1.11 (0.39–3.10)		1 (0.5%)	1 (0.5%)	1.37 (0.08–22.91)	
	Dominant	C/C	172 (65.2%)	156 (60.5%)	1	0.240	141 (69.8%)	120 (57.1%)	1	**0.005**
		T/C-T/T	92 (34.9%)	102 (39.5%)	1.24 (0.86–1.79)		61 (30.2%)	90 (42.9%)	1.82 (1.20–2.77)	
	Recessive	C/C-T/C	256 (97%)	250 (96.9%)	1	0.970	201 (99.5%)	209 (99.5%)	1	0.960
		T/T	8 (3%)	8 (3.1%)	1.02 (0.37–2.83)		1 (0.5%)	1 (0.5%)	1.08 (0.07–17.89)	
	Log-additive	–-	–-	–-	1.18 (0.86–1.63)	0.300	–-	–-	1.78 (1.18–2.68)	**0.006**
rs3751592	Genotype	T/T	198 (74.7%)	210 (77.5%)	1	0.068	170 (84.2%)	164 (75.2%)	1	**0.032**
		C/T	63 (23.8%)	51 (18.8%)	0.73 (0.47–1.12)		30 (14.8%)	49 (22.5%)	1.87 (1.12–3.15)	
		C/C	4 (1.5%)	10 (3.7%)	2.65 (0.80–8.73)		2 (1%)	5 (2.3%)	2.66 (0.50–14.21)	
	Dominant	T/T	198 (74.7%)	210 (77.5%)	1	0.390	170 (84.2%)	164 (75.2%)	1	**0.010**
		C/T-C/C	67 (25.3%)	61 (22.5%)	0.84 (0.56–1.26)		32 (15.8%)	54 (24.8%)	1.93 (1.16–3.19)	
	Recessive	T/T-C/T	261 (98.5%)	261 (96.3%)	1	0.071	200 (99%)	213 (97.7%)	1	0.290
		C/C	4 (1.5%)	10 (3.7%)	2.83 (0.86–9.30)		2 (1%)	5 (2.3%)	2.35 (0.44–12.51)	
	Log-additive	–-	–-	–-	0.97 (0.69–1.38)	0.870	–-	–-	1.81 (1.15–2.86)	**0.009**
rs59429575	Genotype	C/C	190 (72%)	196 (72.3%)	1	0.260	158 (78.2%)	149 (68.3%)	1	**0.047**
		T/C	68 (25.8%)	62 (22.9%)	0.86 (0.57–1.30)		38 (18.8%)	58 (26.6%)	1.71 (1.06–2.76)	
		T/T	6 (2.3%)	13 (4.8%)	2.00 (0.73–5.50)		6 (3%)	11 (5%)	1.99 (0.71–5.64)	
	Dominant	C/C	190 (72%)	196 (72.3%)	1	0.820	158 (78.2%)	149 (68.3%)	1	**0.014**
		T/C-T/T	74 (28%)	75 (27.7%)	0.96 (0.65–1.41)		44 (21.8%)	69 (31.6%)	1.75 (1.11–2.75)	
	Recessive	C/C-T/C	258 (97.7%)	258 (95.2%)	1	0.140	196 (97%)	207 (95%)	1	0.280
		T/T	6 (2.3%)	13 (4.8%)	2.08 (0.76–5.67)		6 (3%)	11 (5%)	1.75 (0.62–4.91)	
	Log-additive	–-	–-	–-	1.05 (0.76–1.46)	0.760	–-	–-	1.57 (1.08–2.29)	**0.016**
Gender	**Males**						**Females**			
rs28757157	Genotype	C/C	213 (65.5%)	192 (60.2%)	1	0.280	100 (70.9%)	84 (56.4%)	1	0.100
		T/C	104 (32%)	120 (37.6%)	1.30 (0.93–1.81)		40 (28.4%)	63 (42.3%)	1.75 (1.03–2.97)	
		T/T	8 (2.5%)	7 (2.2%)	0.91 (0.32–2.60)		1 (0.7%)	2 (1.3%)	1.91 (0.17–21.86)	
	Dominant	C/C	213 (65.5%)	192 (60.2%)	1	0.150	100 (70.9%)	84 (56.4%)	1	**0.033**
		T/C-T/T	112 (34.5%)	127 (39.8%)	1.27 (0.92–1.76)		41 (29.1%)	65 (43.6%)	1.76 (1.04–2.96)	
	Recessive	C/C-T/C	317 (97.5%)	312 (97.8%)	1	0.720	140 (99.3%)	147 (98.7%)	1	0.710
		T/T	8 (2.5%)	7 (2.2%)	0.83 (0.29–2.35)		1 (0.7%)	2 (1.3%)	1.56 (0.14–17.69)	
	Log-additive	–-	–-	–-	1.20 (0.89–1.61)	0.230	–-	–-	1.70 (1.03–2.80)	**0.036**
Smoking	**Yes**						**No**			
rs28757157	Genotype	C/C	164 (69.8%)	140 (59.8%)	1	0.095	149 (64.5%)	136 (58.1%)	1	0.330
		T/C	67 (28.5%)	89 (38%)	1.56 (1.04–2.34)		77 (33.3%)	94 (40.2%)	1.35 (0.91–2.00)	
		T/T	4 (1.7%)	5 (2.1%)	1.37 (0.35–5.28)		5 (2.2%)	4 (1.7%)	0.97 (0.24–3.90)	
	Dominant	C/C	164 (69.8%)	140 (59.8%)	1	**0.031**	149 (64.5%)	136 (58.1%)	1	0.150
		T/C-T/T	71 (30.2%)	94 (40.2%)	1.55 (1.04–2.30)		82 (35.5%)	98 (41.9%)	1.33 (0.90–1.96)	
	Recessive	C/C-T/C	231 (98.3%)	229 (97.9%)	1	0.830	226 (97.8%)	230 (98.3%)	1	0.840
		T/T	4 (1.7%)	5 (2.1%)	1.16 (0.30–4.42)		5 (2.2%)	4 (1.7%)	0.87 (0.22–3.45)	
	Log-additive	–-	–-	–-	1.46 (1.01–2.10)	**0.042**	–-	–-	1.26 (0.88–1.80)	0.210
Drinking	**Yes**						**No**			
rs28757157	Genotype	C/C	110 (73.8%)	82 (60.7%)	1	**0.033**	203 (64%)	194 (58.3%)	1	0.290
		T/C	38 (25.5%)	52 (38.5%)	2.03 (1.18–3.50)		106 (33.4%)	131 (39.3%)	1.29 (0.94–1.79)	
		T/T	1 (0.7%)	1 (0.7%)	2.46 (0.11–57.00)		8 (2.5%)	8 (2.4%)	1.11 (0.41–3.06)	
	Dominant	C/C	110 (73.8%)	82 (60.7%)	1	**0.009**	203 (64%)	194 (58.3%)	1	0.120
		T/C-T/T	39 (26.2%)	53 (39.3%)	2.04 (1.18–3.51)		114 (36%)	139 (41.7%)	1.28 (0.93–1.76)	
	Recessive	C/C-T/C	148 (99.3%)	134 (99.3%)	1	0.700	309 (97.5%)	325 (97.6%)	1	0.980
		T/T	1 (0.7%)	1 (0.7%)	1.85 (0.08–40.76)		8 (2.5%)	8 (2.4%)	1.01 (0.37–2.76)	
	Log-additive	–-	–-	–-	1.99 (1.17–3.36)	**0.010**	–-	–-	1.23 (0.92–1.63)	0.160
rs3751592	Genotype	T/T	121 (80.7%)	108 (75%)	1	0.330	247 (77.9%)	266 (77.1%)	1	0.064
		C/T	27 (18%)	35 (24.3%)	1.52 (0.83–2.79)		66 (20.8%)	65 (18.8%)	0.89 (0.60–1.31)	
		C/C	2 (1.3%)	1 (0.7%)	0.52 (0.04–6.46)		4 (1.3%)	14 (4.1%)	3.23 (1.04–9.97)	
	Dominant	T/T	121 (80.7%)	108 (75%)	1	0.220	247 (77.9%)	266 (77.1%)	1	0.910
		C/T-C/C	29 (19.3%)	36 (25%)	1.45 (0.80–2.62)		70 (22.1%)	79 (22.9%)	1.02 (0.71–1.48)	
	Recessive	T/T-C/T	148 (98.7%)	143 (99.3%)	1	0.550	313 (98.7%)	331 (95.9%)	1	**0.023**
		C/C	2 (1.3%)	1 (0.7%)	0.47 (0.04–5.92)		4 (1.3%)	14 (4.1%)	3.31 (1.07–10.19)	
	Log-additive	–-	–-	–-	1.33 (0.77–2.31)	0.310	–-	–-	1.14 (0.83–1.56)	0.410
rs59429575	Genotype	C/C	117 (78%)	95 (66%)	1	0.130	231 (73.1%)	250 (72.5%)	1	0.190
		T/C	30 (20%)	44 (30.6%)	1.71 (0.97–3.02)		76 (24.1%)	76 (22%)	0.90 (0.62–1.30)	
		T/T	3 (2%)	5 (3.5%)	2.18 (0.46–10.25)		9 (2.8%)	19 (5.5%)	1.95 (0.86–4.41)	
	Dominant	C/C	117 (78%)	95 (66%)	1	**0.044**	231 (73.1%)	250 (72.5%)	1	0.960
		T/C-T/T	33 (22%)	49 (34%)	1.75 (1.01–3.03)		85 (26.9%)	95 (27.5%)	1.01 (0.71–1.42)	
	Recessive	C/C-T/C	147 (98%)	139 (96.5%)	1	0.420	307 (97.2%)	326 (94.5%)	1	0.084
		T/T	3 (2%)	5 (3.5%)	1.87 (0.40–8.73)		9 (2.8%)	19 (5.5%)	2.00 (0.89–4.51)	
	Log-additive	–-	–-	–-	1.63 (1.01–2.64)	**0.044**	–-	–-	1.10 (0.83–1.46)	0.500

*p* values were calculated from logistic regression with adjustments for age, gender, BMI, smoking, and drinking

Bold values indicate statistical significance (*p* < 0.05)

### Stratification analyses by clinical characteristics

As listed in Table 5, the correlation between *CYP19A1* polymorphisms and lung cancer risk in the different groups (tumor type, LNM, and stage) was assessed. The stratified analysis by tumor type demonstrated a relationship between enhanced risk of squamous cell carcinoma and rs28757157 (dominant: *p* = 0.032, OR = 1.59; and additive: *p* = 0.042, OR = 1.48), while rs3751592 CC genotype was identified as a risk factor for lung adenocarcinoma development (genotype: *p* = 0.011, OR = 3.57; and recessive: *p* = 0.013, OR = 3.84). Regrettably, no significant association between *CYP19A1* polymorphisms and lung cancer risk in the stratification analyses by LNM and tumor stage was observed.

**Table 5:** Stratification analyses by clinical characteristics for the association between *CYP19A1* polymorphisms and the risk of lung cancer

SNPs	Model	Genotype	Control	Adenocarcinoma	OR (95%CI)	*p*	Squamous cell carcinoma	OR (95%CI)	*p*
rs28757157	Genotype	C/C	313 (67.2%)	108 (60%)	1	0.240	70 (56.5%)	1	0.099
		T/C	144 (30.9%)	69 (38.3%)	1.37 (0.95–1.98)		50 (40.3%)	1.60 (1.04–2.46)	
		T/T	9 (1.9%)	3 (1.7%)	0.97 (0.25–3.71)		4 (3.2%)	1.56 (0.45–5.40)	
	Dominant	C/C	313 (67.2%)	108 (60%)	1	0.110	70 (56.5%)	1	**0.032**
		T/C-T/T	153 (32.8%)	72 (40%)	1.35 (0.94–1.94)		54 (43.5%)	1.59 (1.04–2.43)	
	Recessive	C/C-T/C	457 (98.1%)	177 (98.3%)	1	0.820	120 (96.8%)	1	0.690
		T/T	9 (1.9%)	3 (1.7%)	0.86 (0.23–3.27)		4 (3.2%)	1.29 (0.38–4.41)	
	Log-additive	–-	–-	–-	1.27 (0.91–1.77)	0.160	–-	1.48 (1.02–2.15)	**0.042**
rs3751592	Genotype	T/T	368 (78.8%)	152 (81.3%)	1	**0.011**	99 (75%)	1	0.260
		C/T	93 (19.9%)	26 (13.9%)	0.67 (0.41–1.08)		29 (22%)	1.16 (0.71–1.91)	
		C/C	6 (1.3%)	9 (4.8%)	3.57 (1.22–10.42)		4 (3%)	3.08 (0.79–12.04)	
	Dominant	T/T	368 (78.8%)	152 (81.3%)	1	0.440	99 (75%)	1	0.330
		C/T-C/C	99 (21.2%)	35 (18.7%)	0.84 (0.54–1.31)		33 (25%)	1.27 (0.79–2.03)	
	Recessive	T/T-C/T	461 (98.7%)	178 (95.2%)	1	**0.013**	128 (97%)	1	0.130
		C/C	6 (1.3%)	9 (4.8%)	3.84 (1.32–11.17)		4 (3%)	2.99 (0.77–11.64)	
	Log-additive	–-	–-	–-	1.04 (0.72–1.49)	0.850	–-	1.33 (0.87–2.01)	0.190

*p* values were calculated from logistic regression with adjustments for age, gender, BMI, smoking, and drinking

Bold values indicate statistical significance (*p* < 0.05)

### The two SNP interactions associated with lung cancer susceptibility.

AS displayed in Table 6, rs28757157-rs3751592 (*p* < 0.001, OR = 2.03), rs28757157-rs3751591 (*p* < 0.001, OR = 1.75), rs28757157- rs59429575 (*p* < 0.001, OR = 1.55), and (*p* = 0.011, OR = 1.31) were associated with the higher lung cancer susceptibility.

**Table 6:** The two SNP interactions associated with lung cancer susceptibility

SNP1	SNP2	OR	95% CI	*p*
rs28757157	rs3751592	2.03	1.66–2.49	** < 0.001**
rs28757157	rs3751591	1.75	1.43–2.13	** < 0.001**
rs28757157	rs59429575	1.55	1.28–1.88	** < 0.001**
rs3751592	rs3751591	1.16	0.94–1.44	0.159
rs3751592	rs59429575	1.31	1.07–1.61	**0.011**
rs3751591	rs59429575	1.13	0.92–1.38	0.254

Bold values indicate statistical significance (*p* < 0.05)

### MDR analysis

**Fig. 2:**
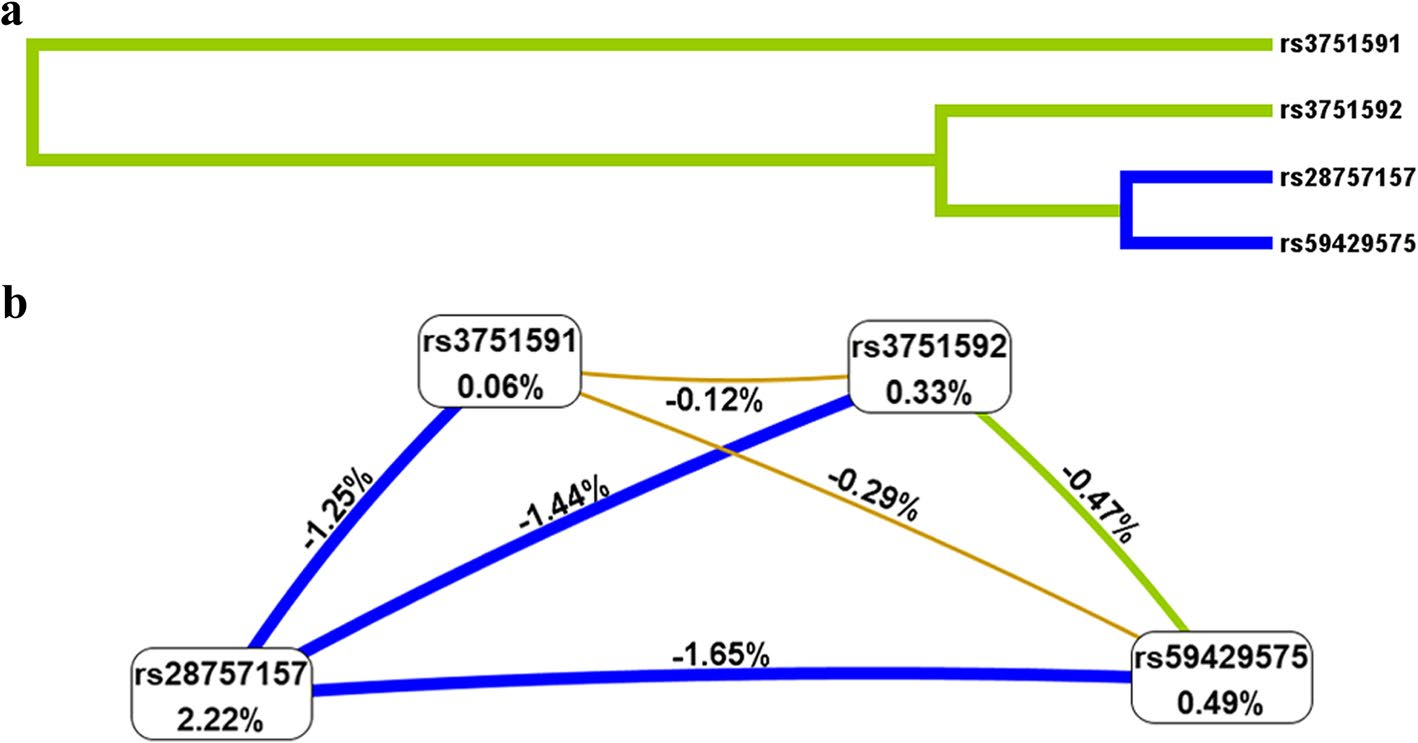
The dendogram (**a**) and Fruchterman Rheingold (**b**) of *CYP19A1* SNP-SNP interaction for the risk of lung cancer. Green and blue color indicated stronger redundant interactions. Values in nodes and between nodes represent the information gains of an individual attribute (main effects) and each pair of attributes (interaction effects), respectively

The association between higher-order SNP–SNP interactions and the predisposition to lung cancer was examined by MDR, as summarized in Fig. 2 and Table 7. Figure 1 presented that these four polymorphisms exhibited strong redundancy effects on the risk of lung cancer, and rs28757157 had the information gain (2.22%) of individual attributes regarding the occurrence of lung cancer. Table 6 summarized that the most influential single-locus attributor for lung cancer risk was rs28757157 (testing balanced accuracy of 0.5503 and cross-validation consistency of 10/10).

**Table 7:** SNP–SNP interaction models of *CYP19A1* polymorphisms in lung cancer susceptibility

Model	Training Bal. Acc	Testing Bal. Acc	CVC	*p*
SNP-SNP interaction				
rs28757157	0.5551	0.5503	10/10	**0.0006**
rs28757157, rs3751592	0.5620	0.5193	4/10	**0.0002**
rs28757157, rs3751592, rs3751591	0.5721	0.5428	9/10	** < 0.0001**
rs28757157, rs3751592, rs3751591, rs59429575	0.5827	0.5450	10/10	** < 0.0001**
*CYP19A1* gene-environment interaction				
rs28757157	0.555	0.550	10/10	**0.0006**
Gender, smoke	0.581	0.581	10/10	** < 0.0001**
Gender, BMI, smoke	0.593	0.560	5/10	** < 0.0001**
rs28757157, rs3751591, gender, smoke	0.608	0.532	5/10	** < 0.0001**
rs28757157, rs3751591, gender, BMI, smoke	0.636	0.601	9/10	** < 0.0001**
rs28757157, rs3751592, rs3751591, gender, BMI, smoke	0.658	0.560	4/10	** < 0.0001**
rs28757157, rs3751592, rs3751591, rs59429575, gender, BMI, smoke	0.685	0.558	8/10	** < 0.0001**

*p* values were calculated using *χ*^2^ tests

Bold values indicate statistical significance (*p* < 0.05)

**Fig. 3:**
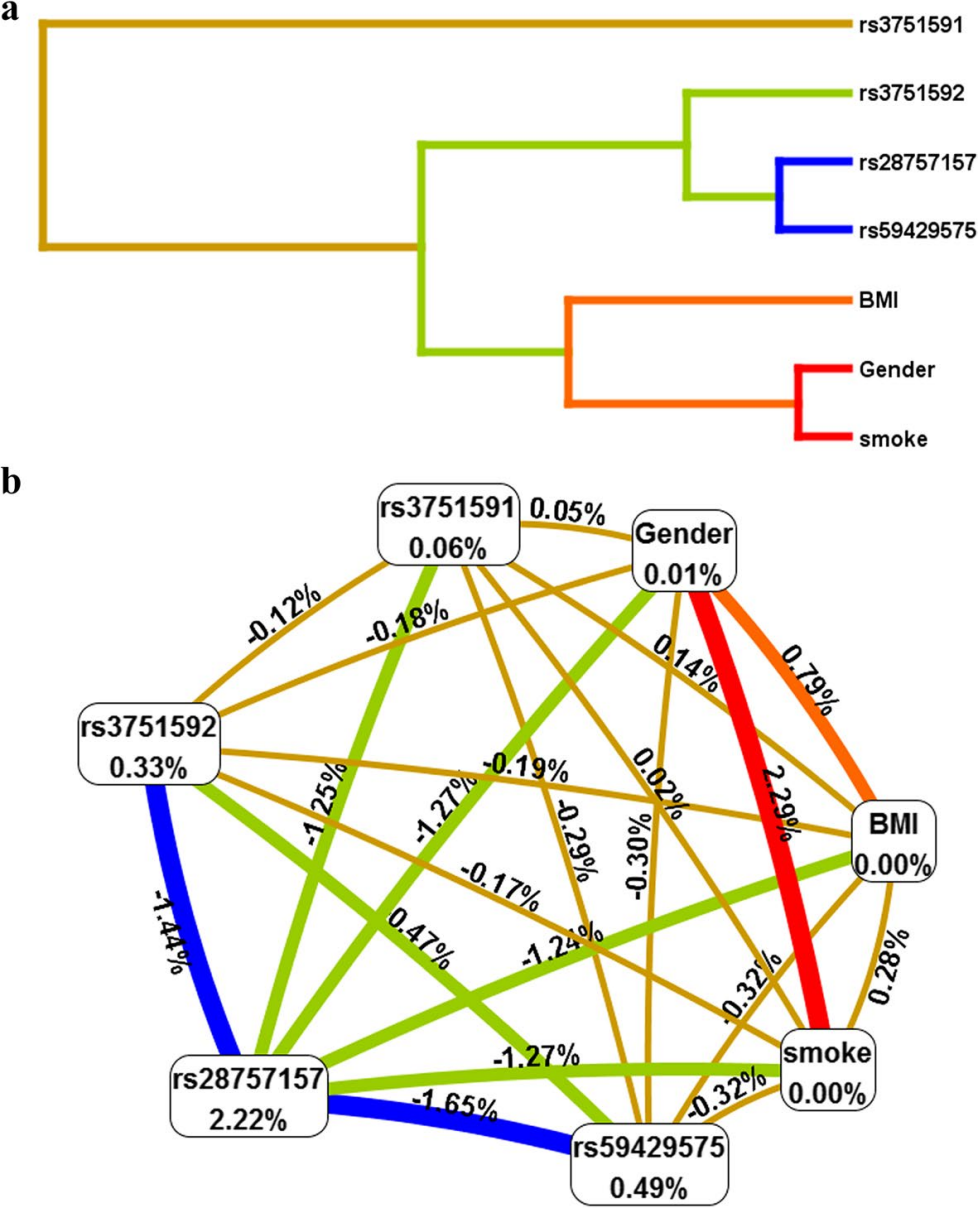
The dendogram (**a**) and fruchterman Rheingold (**b**) of *CYP19A1* gene environment. SNP-SNP interaction for the risk of lung cancer. Green and blue color indicated stronger redundant interactions. Values in nodes and between nodes represent the information gains of individual attributes (main effects) and each pair of attributes (interaction effects), respectively

MDR analysis of gene-environment interaction also suggested that rs28757157 was the most influential single-factor attributor for lung cancer risk. Gender and smoking were found to be the most important environmental factor affecting lung cancer susceptibility. In addition, the gene-environment interaction model, composed of rs28757157, rs3751591, gender, BMI, and smoke showed higher testing-balanced accuracy (0.601) and cross-validation consistency (9/10), indicating that this interaction model was a candidate gene-environment model in our population. Figure 3 exhibited a strong synergy effect of gene-environment interaction on lung cancer risk.

## Discussion

In this study, the association of four SNPs in the *CYP19A1* gene with the susceptibility to lung cancer in the Chinese Han cohort was assessed. Statistical and bioinformatics results highlighted the important roles of rs28757157, rs3751592, and rs59429575 in the outset of lung cancer in the total or stratified population, which helped improve our understanding of *CYP19A1* in this disease.

*CYP19A1* gene, encoding aromatase and responsible for the final step in the biosynthesis of estrogens, estradiol (E2) and estrone (E1), has been intensively investigated [25, 26]. It has been identified that SNPs in the intron region of *CYP19A1* play an important role in the transcriptional regulation and splicing of *CYP19A1* and could produce some different enzymes with diverse enzyme activity compared with normal gene products [27]. The allele frequency of several *CYP19A1* SNPs have been documented in different populations and ethnic groups around the world. SNPs in *CYP19A1* were found to be associated with cancer risk [28]. In particular, *CYP19A1* SNPs have been shown to be significantly associated with lung-related diseases.

A previous study has shown that SNP rs3764221 is significantly correlated with *CYP19A1* expression in non-cancerous lung tissues and affects the susceptibility to lung adenocarcinoma. The authors suggested that *CYP19A1* polymorphisms may lead to elevated levels of local estrogen surrounding the lungs, and this excess local estrogen production may be one of the factors associated with the polycentric development of adenocarcinoma [13]. The recent result has suggested that *CYP19A1* polymorphism is involved in lung bronchioloalveolar carcinoma and atypical adenomatous hyperplasia by causing differences in estrogen levels [29]. It is clear that *CYP19A1* polymorphism may cause changes in estrogen levels around the lungs, which in turn can affect the susceptibility of lung cancer. Our results firstly revealed an association between rs28757157 and increased risk of lung cancer in the genotype, dominant, and additive models. In bioinformatic analysis, results from HaploReg v4.1 database displayed that rs28757157 may be associated with enhancer histone marks, motifs changed, and selected eQTL hits [30]. Based on the QTLbase database, the genotypes of *CYP19A1* rs28757157 (*p* = 6.610e − 5) were related to the mRNA expression of *CYP19A1* in the lungs [31]. These results suggested that *CYP19A1* rs28757157 may be involved in the carcinogenicity of lung cancer by affecting the expression or function of *CYP19A1*, which requires further experimental confirmation.

Notably, the demographic characteristics (age, gender, BMI, smoking, and drinking) might influence the genetic association on the occurrence of lung cancer [32]. Our research showed that *CYP19A1*-rs28757157 was associated with increased cancer risk in the population aged under 60 years, females, smokers, and drinkers. Besides, rs3751592 and rs59429575 were also identified as risk biomarkers in the population aged under 60 years and drinkers. These results indicated that the risk association of these polymorphisms might be age-, sex-, smoking-, and drinking-dependent, and gene-behavioral habit interactions might operate in the pathogenesis of lung cancer.

These SNPs are located in the intron region of the *CYP19A1* gene. Combined with previous studies and database predictions, we speculated that *CYP19A1* intron SNPs may alter mRNA splicing, thereby leading to changes in the activity of *CYP19A1* and related estrogens, and may affect disease susceptibility. Since the statistical significance of the correlation between *CYP19A1* gene polymorphisms and the risk of lung cancer is slightly weak, further experimental studies are needed to verify the results of this study.

Furthermore, the correlation between *CYP19A1* polymorphisms and lung cancer risk in different groups (tumor type, LNM, and stage) was further assessed. Stratified analysis by tumor type demonstrated a relationship between enhanced risk of squamous cell carcinoma and rs28757157, while rs3751592 CC genotype was identified as a risk factor for lung adenocarcinoma development. These findings suggested that lung adenocarcinoma and squamous cell carcinoma may have different genetic pathological mechanisms, which need to be further confirmed.

Our study has several limitations. All subjects were enrolled from the same hospital and the limitations of sample selection may affect the accuracy of this experiment. Subsequently, due to the lack of adequate information on factors such as dietary habits, occupational exposure, and air pollution, this study failed to assess the impact of these factors on the association between *CYP19A1* variants and lung cancer susceptibility. Additional studies that encompass more geographical regions, additional ethnic groups, and larger sample sizes with complete risk factor information should be performed. In order to verify the results of this study, it is necessary to clarify the relationship between the *CYP19A1* gene and lung cancer through subsequent functional studies.

## Conclusions

In summary, our study defined SNPs of *CYP19A1* (rs28757157, rs3751592, and rs59429575), which were significantly associated with lung cancer susceptibility. These variants may be considered as markers for lung cancer risk assessment in the Chinese Han population.

## Supplementary Information

**Additional file 1: Supplementary Figure 1. **The representativespectra of each SNP.

## Data Availability

The datasets generated during the current study are available.

## Abbreviations

CYP450: Cytochrome p450
*CYP19A1*: CYP450 family 19, subfamily A, polypeptide 1
SNPs: Single-nucleotide polymorphisms
BMI: Body mass index
LNM: Lymph node metastasis
