# Supplementary figures and images for: Association of CYP19A1 rs28757157 polymorphism with lung cancer risk in the Chinese Han population
Source: World J Surg Oncol. 2022 Dec 16;20:400. doi: 10.1186/s12957-022-02868-9 (PMC9756459; doi:10.1186/s12957-022-02868-9)

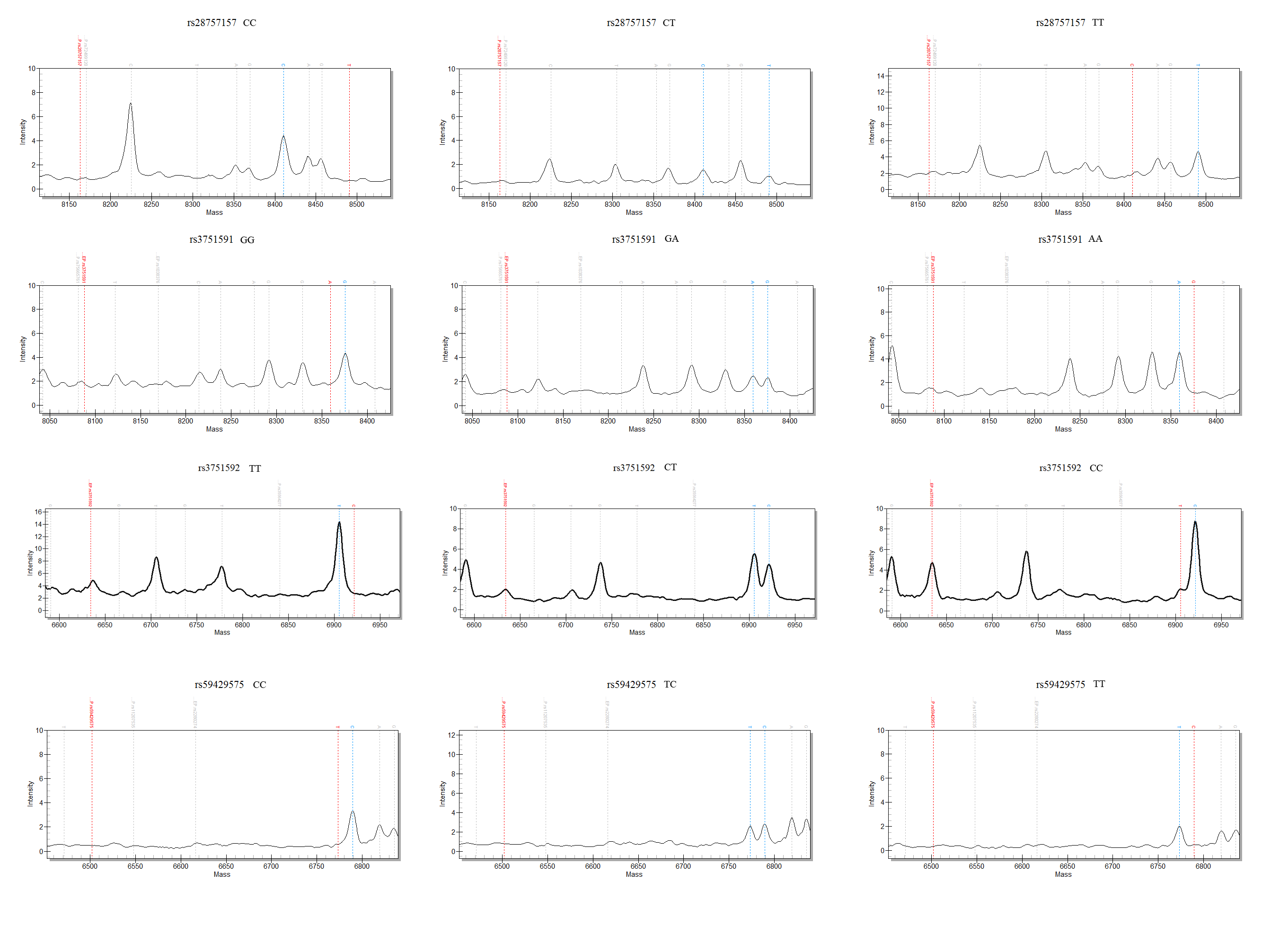

Supplement: Supplementary file 1 — Additional file 1: Supplementary Figure 1. The representativespectra of each SNP. [file 12957_2022_2868_MOESM1_ESM.tif]
